# Is the practice of autonomy support the missing element in teacher training at university? A study on the effects of an intervention based on self-determination theory on biology preservice teachers’ knowledge, beliefs, and intentions

**DOI:** 10.3389/fpsyg.2023.1279771

**Published:** 2023-12-01

**Authors:** Nadine Großmann, Stefan Fries, Matthias Wilde

**Affiliations:** ^1^Department of Biology Education, University of Cologne, Cologne, Germany; ^2^Department of Psychology, Bielefeld University, Bielefeld, Germany; ^3^Department of Biology Didactics, Bielefeld University, Bielefeld, Germany

**Keywords:** autonomy support, teaching behavior, preservice teachers, teacher beliefs, motivation

## Abstract

Teacher education at universities often lacks practice although preservice teachers should be equipped with effective teaching methods. We conducted an intervention to convey and practice autonomy-supportive teaching behavior (ASTB) with 63 preservice teachers. The first control group (*n* = 76) did not receive any information on ASTB. A second control group (*n* = 54) had their regular seminar on ASTB without practicing what they had learned. Significant differences in the participants’ (*M*_age_ = 24.18 ± 3.26 years; *M*_semester_ = 7.61 ± 1.84; 63% female) knowledge, beliefs, and intentions were found. Participants of the intervention had higher theoretical and practical knowledge regarding ASTB and a higher level of perception that ASTB is easy to implement than a seminar without information on ASTB (first control group) and the regular seminar (second control group). That is, their knowledge acquisition and their belief about the ease of implementation were positively affected by the intervention in comparison to both control groups. Although no significant differences were found, preservice teachers in the intervention group also showed the highest level of agreement with the belief about the effectiveness of ASTB and the most pronounced intention to implement ASTB. The results of our study show that the developed intervention based on self-determination theory had a positive impact on preservice teachers’ knowledge and beliefs about ASTB as well as their intention to apply it in the future. Since teacher education at university often lacks practice, the development of such effective theory- and practice-based interventions for preservice teachers is especially important. The practical skills acquired in our study are of particular importance for one central challenge teachers face in their profession: the need to counteract the decreasing trend of student motivation during secondary schooling.

## 1 Introduction

Comprehensive practical phases in teacher education at universities are demanded by both educators and preservice teachers (Grossman et al., 2009; Philip et al., 2019). Such phases promise the acquisition of meaningful teaching skills before entering the teaching profession. In Germany, one consequence of these demands is the integration of a practical semester of six months into preservice teachers’ master’s studies (e.g., MSWNRW, 2009; Weyland and Wittmann, 2017). Besides these practical phases at school, consideration should also be given to how the development of practical skills can be supported at the university level. This is especially important for one central challenge teachers face in their profession: the need to counteract the decreasing trend of student motivation during secondary schooling (Gillet et al., 2012; Scherrer and Preckel, 2019). Unfortunately, teachers often lack the didactic-methodological skills to foster student motivation (Reeve et al., 2004) and tend to exhibit demotivating behaviors (Barrett and Boggiano, 1988; Turner, 2010).

Teaching central concepts of self-determination theory (SDT; Ryan and Deci, 2017) is one way of providing teachers with effective measures to support student motivation in the classroom. These measures are most often oriented toward students’ basic need for autonomy (Reeve, 2015). Students in autonomy-supportive settings experience more self-determined motivation than students who feel controlled (Mittag et al., 2009; Haerens et al., 2015; Froiland et al., 2017; Großmann and Wilde, 2022). Besides these beneficial effects of autonomy support for their students, the concepts and assumptions behind SDT are also relevant to the teachers’ own well-being (e.g., Roth, 2014) and can help them to evaluate and regulate their own motivational orientation. Regulating one’s own motivational orientation is seen as an essential professional competence of teachers (Kunter et al., 2013; Fauth et al., 2019).

Various studies have shown that knowledge- and skill-based interventions can help teachers learn about autonomy-supportive teaching behavior (ASTB) and apply these behaviors in class (Assor et al., 2009; Su and Reeve, 2011; Aelterman et al., 2014, 2016; Cheon and Reeve, 2015; De Naeghel et al., 2016; Reeve and Cheon, 2016; for an overview: Reeve and Cheon, 2021). In particular, these interventions have an impact on the teachers’ beliefs about the ease of implementation and effectiveness of ASTB as well as their intention to apply it (Reeve et al., 2014; Reeve and Cheon, 2016). Such interventions are especially effective when they are implemented in teacher training at university (see Su and Reeve, 2011) since preservice teachers are still flexible in terms of their behavior in class because their teacher personality has not yet been established (Hoy and Woolfolk, 1990; Tessier et al., 2010).

Thus, we designed an intervention based on SDT (Ryan and Deci, 2017) to convey and practice the central concepts of this theory and the ASTB derived from them. We were interested in whether this intervention can have an impact on preservice teachers’ beliefs about ASTB as well as their intention to apply it. Since preservice teachers need the corresponding theoretical and practical knowledge to implement ASTB successfully, we further considered these variables in our study. To test the effectiveness of the intervention, we compared the experimental group with one control group that did not receive any information on SDT and a second control group that had the regular university seminar dealing with ASTB, but did not have the opportunity to practice ASTB.

## 2 Theory

### 2.1 Autonomy and motivation in self-determination theory

As a sub-theory of Ryan and Deci’s (2017) SDT, basic psychological needs theory posits that there are three innate basic psychological needs, namely the need for relatedness, competence, and autonomy. The need for relatedness describes the desire to interact with significant individuals and the desire to belong to a social community (Ryan and Deci, 2017). The need for competence comprises the wish to perceive and develop one’s own ability and effectiveness in his/her actions (Ryan and Deci, 2017). The need for autonomy describes the striving of individuals to perform actions voluntarily and without perceived internal and external pressure (*volition*; Reeve et al., 2003; Reeve, 2015). Furthermore, individuals want to have meaningful choices in their actions (*choice*; Reeve et al., 2003; Reeve, 2015) and want to experience themselves as the origins of these actions (*internal locus of causality*; Reeve et al., 2003; Reeve, 2015).

The qualities of motivation that arise from the satisfaction or frustration of the three depicted needs are described in organismic integration theory (Ryan and Deci, 2017). In this second sub-theory of SDT, Ryan and Deci (2017) distinguish between intrinsic and extrinsic motivation. The goal of intrinsically motivated actions is the execution of the action itself (Vallerand and Ratelle, 2002; Ryan and Deci, 2017). These actions are taken up voluntarily and are characterized by pleasure, spontaneity, and curiosity (Vallerand and Ratelle, 2002; Ryan and Deci, 2017). Extrinsically motivated actions are carried out due to external incentives that can be separated from the action (Vallerand and Ratelle, 2002; Ryan and Deci, 2017). Four types of regulation can underlie extrinsically motivated actions: external, introjected, identified, and integrated (Vallerand and Ratelle, 2002; Ryan and Deci, 2017). These types of regulation can be arranged on a continuum of self-determination (Ryan and Deci, 2017). External regulation represents the most heteronomous (externally determined) and integrated regulation is the most autonomous (self-determined) type of regulation (Vallerand and Ratelle, 2002; Ryan and Deci, 2017).

The concepts and assumptions behind SDT (Ryan and Deci, 2017) are not only important for supporting students’ perception of autonomy and self-determined motivation in class (Mittag et al., 2009; Haerens et al., 2015; Froiland et al., 2017; Großmann and Wilde, 2022), but they are also of personal relevance for the pre- and in-service teachers themselves. The aforementioned basic needs play a central role in teachers’ well-being and their quality of motivation in the teaching profession (Roth et al., 2007; Roth, 2014; Ryan and Deci, 2020). Furthermore, knowledge about one’s own motivational quality and about possibilities for satisfying one’s own psychological needs can enable individuals to evaluate and regulate their own motivational orientation. A teacher’s motivational orientation can have an impact on the motivation experienced by the students he or she teaches (Müller et al., 2009; Ryan and Deci, 2020). This explains, among other things, why the skills to evaluate and regulate one’s own motivational regulation are described as important components of the professional competence of teachers (Kunter et al., 2013; Fauth et al., 2019).

To reflect on both their own and their students’ motivation, an intervention that conveys the concepts and assumptions behind the depicted sub-theories of SDT was designed for preservice teachers. Since teachers often lack practical skills to foster student motivation (Reeve et al., 2004), the communication and practice of ASTB anchored in SDT is particularly important in such interventions. ASTB and the contrasting controlling teaching behavior (CTB) are focused on in the following section.

### 2.2 Autonomy support and control in the sense of self-determination theory

The students’ perception of autonomy and their motivation can be affected by the teacher’s behavior in the classroom (Assor et al., 2002; Reeve, 2015). CTB is often observed in the classroom, in particular among preservice teachers and teachers who experience large amounts of pressure at work (Barrett and Boggiano, 1988; Leroy et al., 2007; Turner, 2010; Ryan and Deci, 2020). The negative effects of this behavior on students’ self-determined motivation have been confirmed in various studies (Assor et al., 2005; De Meyer et al., 2014; Großmann and Wilde, 2022). CTB is characterized, among other things, by the frequent use of commands as well as statements with the phrase ‘You shall...’ or ‘You must...’ (Reeve, 2015; Reeve and Cheon, 2021). Controlling teachers put their students under (time) pressure, motivate them with external incentives such as punishment, rewards, or grades, and give controlling feedback (Deci, 1971; Ryan, 1982; Reeve et al., 1999; Reeve, 2015). In controlling feedback, social comparisons and the teacher’s expectations are used to evaluate student performance (Ryan, 1982; Kast and Connor, 1988; Katz and Assor, 2007; Ryan and Deci, 2017). This type of feedback creates pressure to exhibit expected behavior (Ryan, 1982; Ditton and Müller, 2014). Lastly, controlling teachers do not provide choices or rationales for the topics and actions in class (Reeve, 2015).

On the other hand, teacher behavior that supports students’ autonomy is characterized by the consideration of negative feelings, the appreciation of student wishes, ideas, and opinions, and gives students the freedom to work autonomously (Su and Reeve, 2011; Reeve, 2015; Reeve and Cheon, 2021). A respectful and appreciative attitude toward the students is especially important here (Reeve, 2002, 2009; Reeve and Jang, 2006). Autonomy-supportive teachers grant their students freedom of choice and provide informative feedback that appreciates student performance and advises on the further learning process (Katz and Assor, 2007; Carpentier and Mageau, 2013; Reeve, 2015). In this kind of feedback (as well as in the classroom), neutral language is used that allows flexibility in subsequent behavior and is characterized by statements such as ‘You can...’ or ‘If you want to...’ (see Su and Reeve, 2011). Moreover, autonomy-supportive teachers show students the usefulness and personal relevance of their actions and the topics in class (Su and Reeve, 2011; Reeve and Cheon, 2021).

The depicted ASTBs have been shown to have a positive effect on students’ quality of motivation (Mittag et al., 2009; Haerens et al., 2015; Froiland et al., 2017; Großmann and Wilde, 2022) and can be learned in interventions (Reeve, 1998; Assor et al., 2009; Su and Reeve, 2011; Aelterman et al., 2014; Cheon and Reeve, 2015; De Naeghel et al., 2016; Reeve and Cheon, 2016; for an overview: Reeve and Cheon, 2021). However, in addition to sufficient theoretical and practical knowledge, the intention to implement as well as an actual application of ASTB depends on the teachers’ beliefs about its ease of implementation, effectiveness, and normalcy (Roth and Weinstock, 2013; Reeve and Cheon, 2016; Tan and Levesque-Bristol, 2023). These beliefs are discussed in the following section.

### 2.3 Teachers’ beliefs about autonomy-supportive teaching behavior

Reeve et al. (2014) assume three generalized beliefs about ASTB that are essential for the implementation of this teaching behavior. The first belief regarding the ease of implementation describes whether a teacher thinks a didactic approach is easy or difficult to implement in his/her instruction (Reeve et al., 2014; Reeve and Cheon, 2016). The second belief deals with the perceived effectiveness of a didactic approach and indicates whether a teacher thinks it is overall effective in motivating and activating his/her students (Reeve et al., 2014; Reeve and Cheon, 2016). Third, the normalcy belief describes the extent to which a teacher perceives a didactic approach as the norm in his/her personal school context, that is, whether it is accepted, expected, and implemented in his/her environment (Reeve et al., 2014; Reeve and Cheon, 2016). Since teachers are usually convinced that ASTB is difficult to implement and ineffective (Turner et al., 2011; Reeve and Cheon, 2016), special consideration needs to be given to these beliefs in interventions about this teaching behavior. The third belief about the normalcy of ASTB was not considered in the current study because preservice teachers do not yet belong to a specific school environment in which they would perceive these norms (see Reeve et al., 2014). Moreover, this belief about the school environment presumably cannot be influenced by interventions at university.

The investigation of previous interventions based on SDT reveals that these interventions can affect the depicted beliefs as well as the participants’ knowledge, intention, and behavior (Aelterman et al., 2014; Reeve and Cheon, 2016; Assor et al., 2018; Tilga et al., 2021). However, a change in beliefs and behavior can only be expected if teachers recognize the relevance, ease of implementation, and effectiveness of ASTB (Su and Reeve, 2011; De Naeghel et al., 2016; Reeve and Cheon, 2016).

To foster positive beliefs about ASTB and equip preservice teachers with an effective method to support their students’ motivation in the classroom, we developed an intervention dealing with the two sub-theories of SDT (basic psychological needs and organismic integration theory) and ASTB. We investigated the following hypotheses to evaluate the effectiveness of this intervention.

## 3 Hypotheses

Three groups were investigated in the current study. In the experimental group, the two depicted sub-theories of SDT as well as ASTB were conveyed, practiced, and discussed. The first control group did not receive any information on SDT. The second control group attended a regular seminar in which the sub-theories and ASTB were conveyed and discussed, but not practiced.

The preservice teachers in the experimental group exhibit a higher degree of theoretical and practical knowledge about ASTB…

H1a …than the preservice teachers in the first control group.

H1b …than the preservice teachers in the second control group.

The preservice teachers in the experimental group believe ASTB to be easier to implement…

H2a …than the preservice teachers in the first control group.

H2b …than the preservice teachers in the second control group.

The preservice teachers in the experimental group believe ASTB to be more effective…

H3a …than the preservice teachers in the first control group.

H3b …than the preservice teachers in the second control group.

The preservice teachers in the experimental group report a higher degree of intention to implement ASTB…

H4a …than the preservice teachers in the first control group.

H4b …than the preservice teachers in the second control group.

## 4 Methods

### 4.1 Sample

In Germany, teacher training at the tertiary level consists of a bachelor’s (six semesters) and a master’s degree (four semesters). The practical semester at school is embedded in the second master’s semester. The 193 biology preservice teachers in our study were in seminars that had prepared them for their practical semester and therefore mainly in the first semester of their master’s studies (*M*_semester_ = 7.61, *SD*_semester_ = 1.84, *Mdn*_semester_ = 8.00; 63% female). On average, these preservice teachers were 24.18 years old (*SD* = 3.26 years, *Mdn* = 23.00 years). 37.4% of the participants studied a second STEM subject besides biology, whereas 19.1% of them were in their training for a second subject in the field of languages. 15.4% of the investigated preservice teachers reported being trained in a social second subject, and 28.1% of them took physical education as a second subject in their studies. Sixty-three preservice teachers were part of the experimental group (EG). The first control group (CG 1) was composed of 76 preservice teachers, whereas 54 preservice teachers were assigned to the second control group (CG 2).

The current study was conducted in an ecologically valid context of the actual teacher education at the German university where the investigation was conducted. Within this context, a randomized distribution of participants to the different groups was not possible. Preservice teachers chose different seminars and were asked afterward to participate in the current study (cluster sampling). Our criteria for the sampling were that the participants were in their master’s studies and trained to teach biology at the secondary level. In three semesters, all preservice teachers at the investigated university who met these criteria were examined.

### 4.2 Study design

The present study is a quasi-experimental study with a pretest-posttest control group design. One week before the intervention, a pretest was conducted in all three investigated groups. The pretest assessed the preservice teachers’ knowledge and beliefs about ASTB as well as their intention to apply it (see Section 4.3). The following two weeks differed between the groups. The EG received an intervention based on SDT that was composed of three 90-min seminar sessions (see Section 4.4; Großmann et al., 2019). CG 1 received no intervention and participated in a seminar to prepare them for their practical semester in which no content on SDT was covered. CG 2 had the regular seminar as well but learned about SDT in two 90-min seminar sessions. In the first session, the concepts and assumptions behind SDT were conveyed and discussed. In the second seminar session, ASTB was conveyed and discussed based on empirical investigations of this teaching behavior. The basic content in CG 2 was identical to the content of the intervention. However, students in the experimental group received further information on ASTB such as example operationalizations in the practical seminar sessions which they needed to practice the content. After these two weeks, the posttest was conducted in the three groups within one week. This posttest examined the same variables that were assessed in the pretest. Additionally, the preservice teachers’ perceived autonomy during the seminar sessions was surveyed.

We chose to implement two control groups for the following reason: The first control group was used to check whether the students might have been exposed to the contents of the intervention in other seminars during the time of the intervention. The second control group was needed to test whether the intervention with opportunities to practice the content had positive effects on the studied constructs compared to the regular seminar on SDT and ASTB without practice.

### 4.3 Test instruments

#### 4.3.1 Knowledge tests

The preservice teachers’ knowledge about ASTB was assessed with self-developed tests in the pre- and posttest. Their theoretical knowledge was evaluated with seven open-ended items (example item: “Define the need for autonomy.”), while eight open-ended items examined their practical knowledge (example item: “Give two examples of choices that you can offer your students.”). Each open-ended item was rated with either zero, one, or two points. The preservice teachers received zero points for not answering or for giving an incorrect answer. One point was given for a partially correct answer. The preservice teachers received two points for a correct and complete answer. For the items on both knowledge tests, an excellent interrater agreement for the two raters of the items was found (theoretical knowledge: Cohen’s κ = 0.91; practical knowledge: Cohen’s κ = 0.93). Fifteen percent of the tests were rated twice to determine interrater agreement.

#### 4.3.2 Teaching scenarios measure

The preservice teachers’ beliefs about the ease of implementation and the effectiveness of ASTB as well as their intention to apply it were investigated with the *Teaching Scenarios Measure* (Reeve et al., 2014) in the pre- and posttest. This test instrument contains a written scenario that depicts ASTB without naming or labeling it. On a five-point rating scale (‘0 = strongly disagree’ to ‘4 = strongly agree’), the items were rated with regard to the scenario. The number of items as well as example items are shown in Table 1. Internal consistency for the scales ranged from satisfying to excellent (Table 1).

**Table 1:** Internal consistencies and example items for the Teaching Scenarios Measure and the Learning Climate Questionnaire.

Test instrument	Construct	Example item	Cronbach’s Alpha
Teaching Scenarios Measure (Reeve et al., 2014)			
	*Belief about the ease of implementation* *(four items)*	This approach to teaching is effortless and easily manageable.	α_pre_ = 0.88; α_post_ = 0.93
	*Belief about the effectiveness* *(four items)*	In terms of performance and achievement, students benefit from this approach to teaching.	α_pre_ = 0.74; α_post_ = 0.78
	*Intention* *(four items)*	I plan to teach my students this way in the future.	α_pre_ = 0.84; α_post_ = 0.75
Learning Climate Questionnaire (Black and Deci, 2000)	*Perceived autonomy* *(nine items)*	My instructor listens to how I would like to do things.	α = 0.89

#### 4.3.3 Learning climate questionnaire

The preservice teachers’ perceived degree of autonomy during the intervention and their regular seminar, respectively, was assessed with the *Learning Climate Questionnaire* (Black and Deci, 2000) in the posttest. The same five-point rating scale was used to rate the nine items of this questionnaire. An example item can be found in Table 1. The Cronbach’s alpha value for the items was satisfying (Table 1).

### 4.4 Design of the intervention

The design of the intervention in the EG was based on the findings of recent studies and meta-analyses of interventions conducted according to SDT (Su and Reeve, 2011; Ryan and Deci, 2017). Among other things, previous studies revealed that knowledge- and skill-based interventions that do not exceed three hours per session and apply different types of media are particularly effective (Su and Reeve, 2011; De Naeghel et al., 2016). Therefore, audio and video sequences, tablets or laptops, smartphones, as well as paper-and-pencil-based tasks were used in all three seminar sessions. The knowledge- and skill-based intervention was composed of two parts. The first part consisted of one 90-min seminar session on basic psychological needs theory and organismic integration theory (see Section 2.1). In this session, the central concepts and assumptions behind these sub-theories were conveyed, practiced, and discussed. For example, the preservice teachers described students’ psychological needs satisfaction in frequently occurring situations in the classroom or searched for actions based on the different motivational regulations in a self-written story about internalization processes. In the second part, the preservice teachers learned about, practiced, and discussed ASTB in two 90-min practical seminar sessions. Five ASTBs were dealt with in these sessions: acknowledging negative feelings, providing rationales, offering choices, giving informative feedback, and using neutral language (see Section 2.2). The preservice teachers learned about and practiced these behaviors at various stations where they worked in small groups. For example, they designed rationales for topics and actions in biology lessons, analyzed videos of different teaching behaviors in class, and performed role plays on students’ negative feelings. The practiced behaviors were reflected on and discussed regarding their application in the classroom at the end of each session.

As instrumental support and follow-up activities are further characteristics of an effective intervention (Assor et al., 2009; Su and Reeve, 2011), the preservice teachers received (1) a reader dealing with the theoretical and empirical background of SDT (Ryan and Deci, 2017) and ASTB, (2) a booklet on ASTB and its application with practical examples, and (3) a glossary with important concepts and assumptions behind SDT (Ryan and Deci, 2017). In addition, a self-developed observation grid based on the *Learning Climate Questionnaire* (Black and Deci, 2000) was given to the preservice teachers, which was designed to guide their observation of ASTB and CTB in the classroom as a follow-up activity.

Besides the aforementioned characteristics, previous studies show that interventions should consider the participants’ basic need satisfaction during the intervention (Assor et al., 2009; De Naeghel et al., 2016). Therefore, the instructor of the intervention behaved in an autonomy-supportive way in the three sessions. At the beginning of the intervention, the preservice teachers were provided with a rationale that showed them the personal relevance and practical value of SDT (Ryan and Deci, 2017) and the autonomy support anchored therein. For this purpose, current findings on students’ quality of motivation were used to show why preservice teachers should know and be able to apply theories and behaviors to foster student motivation. Furthermore, the importance of their own need satisfaction and quality of motivation in the teaching profession was highlighted. Freedom of choice was first granted regarding the group size and membership in the group work phases during the intervention. In addition, the preservice teachers were able to decide how much time they wanted to spend and the order in which they wanted to work at the stations. If there were different materials to choose from at the stations, the preservice teachers were allowed to decide which materials they wanted to work with. In line with autonomy support in the classroom, the instructor acknowledged and accepted negative feelings, which occur frequently when discussing ASTB (see Turner et al., 2011; Reeve and Cheon, 2016). In addition, the preservice teachers’ statements as well as their work were valued and their perspectives, ideas, and wishes were taken into account. When the preservice teachers asked for feedback, the instructor designed it to be informative and used neutral language, as he/she did in all communication in the intervention.

### 4.5 Statistics

To compare the preservice teachers’ perceived degree of autonomy in the three groups, we first applied a univariate analysis of variance. Second, analyses of variance with repeated measures were conducted to investigate the preservice teachers’ knowledge, beliefs, and intention to use ASTB in the three groups. Since analyses of variance are omnibus tests (Field, 2016), simple contrasts were applied in the last step to examine possible differences between the EG and CG 1 as well as between the EG and CG 2. To determine the effect size of the contrasts, we used Pearson’s *r* (e.g., Rosnow et al., 2000).

## 5 Results

Preliminary, the preservice teachers’ perceived degree of autonomy during their seminars was investigated. Analysis of variance showed that the three groups differed significantly regarding their perception of autonomy [*F*(1, 190) = 14.28, *p* < 0.001, η^2^ = 0.13]. Simple contrasts revealed significant differences between the EG and CG 1 [*t* (190) = 3.82, *p* < 0.001, *r* = 0.31] as well as between the EG and CG 2 [*t* (190) = 5.14, *p* < 0.001, *r* = 0.47] with medium effect sizes. The preservice teachers in the EG (*M* = 3.64, *SD* = 0.37) perceived a higher degree of autonomy than the preservice teachers in CG 1 (*M* = 3.32, *SD* = 0.55) and CG 2 (*M* = 3.17, *SD* = 0.51).

With regard to our hypotheses, the preservice teachers’ knowledge regarding ASTB was surveyed afterward (Table 2). Significant interaction effects of the factors time and treatment with a large effect size were revealed by the analyses of variance with repeated measures for their theoretical knowledge [main effect time: *F*(1, 190) = 290.83, *p* < 0.001, η^2^ = 0.61; main effect treatment: *F*(1, 190) = 30.55, *p* < 0.001, η^2^ = 0.24; time x treatment: *F*(1, 190) = 73.38, *p* < 0.001, η^2^ = 0.44] and their practical knowledge [main effect time: *F*(1, 190) = 220.02, *p* < 0.001, η^2^ = 0.54; main effect treatment: *F*(1, 190) = 36.54, *p* < 0.001, η^2^ = 0.28; time x treatment: *F*(1, 190) = 126.45, *p* < 0.001, η^2^ = 0.57]. Descriptively, the largest difference between pretest and posttest knowledge can be found in the EG, whereas smaller differences can be found for CG 1 and CG 2. The following simple contrasts revealed significant differences between the EG and both CGs for both knowledge dimensions (Table 2). In all comparisons, the preservice teachers in the EG had a higher degree of theoretical and practical knowledge after the intervention than the respective control group. Medium to large effect sizes were found for these comparisons, with larger effect sizes for the EG and CG 1 comparison than for the EG and CG 2 comparison.

**Table 2:** Means and standard deviations of all investigated variables in the experimental group (EG), the first control group (CG 1) and the second control group (CG 2) as well as the simple contrasts for the comparison of these groups.

	EG		CG 1		CG 2		Simple contrasts	
	Pretest	Posttest	Pretest	Posttest	Pretest	Posttest		
	*M* (*SD*)	*M* (*SD*)	*M* (*SD*)	*M* (*SD*)	*M* (*SD*)	*M* (*SD*)	EG and CG 1	EG and CG 2
Theoretical knowledge	1.10 (1.57)	4.06 (1.72)	0.64 (0.94)	1.01 (1.28)	1.31 (1.50)	2.69 (1.92)	*t* (190) = 7.61 *p* < 0.001 *r* = 0.71	*t* (190) = 2.30 *p* = 0.022 *r* = 0.35
Practical knowledge	4.21 (2.16)	10.93 (2.43)	4.56 (2.01)	5.26 (1.89)	5.26 (2.92)	5.86 (2.13)	*t* (190) = 8.36 *p* < 0.001 *r* = 0.80	*t* (190) = 5.77 *p* < 0.001 *r* = 0.74
Belief about the ease of implementation	1.45 (0.65)	2.33 (0.80)	1.52 (0.79)	1.62 (0.70)	1.53 (0.69)	1.76 (0.71)	*t* (190) = 3.04 *p* = 0.003 *r* = 0.43	*t* (190) = 2.17 *p* = 0.032 *r* = 0.35
Belief about the effectiveness	2.91 (0.48)	3.35 (0.52)	3.00 (0.49)	3.10 (0.47)	3.05 (0.50)	3.29 (0.51)	*t* (190) = 1.18 *p* = 0.240 *r* = 0.26	*t* (190) = 0.51 *p* = 0.607 *r* = 0.06
Intention	2.92 (0.66)	3.36 (0.60)	3.01 (0.57)	3.05 (0.59)	3.03 (0.61)	3.15 (0.55)	*t* (190) = 1.19 *p* = 0.236 *r* = 0.25	*t* (190) = 0.49 *p* = 0.627 *r* = 0.18

Regarding the preservice teachers’ beliefs about ASTB, the analysis of variance with repeated measures showed significant interaction effects with a medium to large effect size for their beliefs about the ease of implementation [main effect time: *F*(1, 190) = 48.44, *p* < 0.001, η^2^ = 0.20; main effect treatment: *F*(1, 190) = 17.93, *p* = 0.009, η^2^ = 0.05; time x treatment: *F*(1, 190) = 17.93, *p* < 0.001, η^2^ = 0.16] and the effectiveness of ASTB [main effect time: *F*(1, 190) = 50.29, *p* < 0.001, η^2^ = 0.21; main effect treatment: *F*(1, 190) = 1.51, *p* = 0.223, η^2^ = 0.02; time x treatment: *F*(1, 190) = 8.17, *p* < 0.001, η^2^ = 0.08]. The descriptive data show that the difference between pretest and posttest values for the belief about the ease of implementation in the EG is larger than in CG 1 and CG 2 (Table 2). Regarding this belief, an analysis with simple contrasts revealed significant differences for both comparisons with a medium effect size. In comparison to CG 1 and CG 2, the preservice teachers in the EG perceived ASTB to be easier to implement after the intervention than the preservice teachers in CG 1 and CG 2. However, preservice teachers in the EG attributed similar levels of effectiveness to ASTB after the intervention when compared to the preservice teachers in CG 1 and CG 2. An analysis with simple contrasts confirmed that there were no significant differences between the EG and the two control groups. If the pretest values are taken into account, the largest difference between the reported beliefs about the effectiveness in the pretest and posttest can be found descriptively in the EG.

Lastly, a significant interaction effect of the factors time and treatment with a medium effect size became evident for the preservice teachers’ intention to apply ASTB [main effect time: *F*(1, 190) = 21.05, *p* < 0.001, η^2^ = 0.10; main effect treatment: *F*(1, 190) = 0.72, *p* = 0.490, η^2^ = 0.01; time x treatment: *F*(1, 190) = 7.87, *p* < 0.001, η^2^ = 0.08]. Descriptively, the largest difference between the reported intention to use ASTB in the pretest and posttest was found in the EG, while these differences were smaller for CG 1 and CG 2 (Table 2). However, significant differences between the EG and both CGs could not be found in the analysis with simple contrasts. The preservice teachers in the EG, CG 1, and CG 2 reported a similar degree of intention to apply ASTB after the intervention.

## 6 Discussion

In the current study, we investigated whether an intervention based on SDT (Ryan and Deci, 2017) would have a positive effect on preservice teachers’ knowledge and beliefs about ASTB as well as their intention to apply it in the future. To test these effects, we compared the values of these variables in the EG with the ones in two control groups: CG 1, which did not receive any content related to SDT, and CG 2, which took part in a regular seminar in which the central concepts and assumptions behind SDT as well as ASTB were conveyed and discussed, but not practiced. As a preliminary result, we found that the preservice teachers in the EG, in which the instructor behaved in an autonomy-supportive way, reported a higher perception of autonomy than the preservice teachers in CG 1 and CG 2 did. We therefore assume that the implementation of this behavior during the intervention was successful.

### 6.1 Theoretical and practical knowledge

Regarding the preservice teachers’ theoretical and practical knowledge, we found that the preservice teachers in the EG had the highest level of theoretical and practical knowledge in comparison to the preservice teachers in CG 1 (H1a) and CG 2 (H1b). For the comparison between the EG and CG 1, this finding is hardly surprising. Much more remarkable is that teaching central concepts and assumptions behind SDT as well as ASTB seems to have been more successful in the intervention than in the preservice teachers’ regular seminar, which dealt with the same content but lacked practice. The practice of SDT content and ASTB during the intervention seems to have had an additional effect on the preservice teachers’ acquisition of theoretical and practical knowledge, for it might have allowed them to deepen the conveyed content. Such practical phases have also been implemented in previous studies that found positive effects of interventions based on SDT on teachers’ beliefs about ASTB (e.g., Aelterman et al., 2014; Reeve and Cheon, 2016). We discuss these studies in more detail throughout the discussion. In addition to the practical phases during the intervention, the instructor’s autonomy support may have had an impact on their quality of motivation and, consequently, their knowledge acquisition (see Black and Deci, 2000; Kaplan and Madjar, 2017; Ryan and Deci, 2017; Gutiérrez and Tomás, 2019).

In addition to the significant differences between the three groups, a descriptive comparison shows that the preservice teachers in all groups had more practical than theoretical knowledge at the beginning of the study. The preservice teachers might have acquired this knowledge in previous seminars related to didactics during their bachelor studies. However, the preservice teachers did not seem to have learned about the theoretical concepts and assumptions behind SDT in previous seminars. In this discussion of the preservice teachers’ theoretical and practical knowledge acquisition, it should be noted that previous studies on interventions based on SDT did not examine the participants’ pre- and posttest knowledge, or only indirectly examined it, for example by observing its use in the classroom (e.g., Aelterman et al., 2014; for an overview: Reeve and Cheon, 2021). Thus, comparisons with previous studies are currently not possible.

### 6.2 Beliefs about the ease of implementation and effectiveness

With regard to the preservice teachers’ belief about the ease of implementation of ASTB, we again found significant differences between the EG and both CGs (H2a, H2b). This result is in line with previous studies, which show that interventions based on SDT have an impact on the participants’ beliefs about ASTB (Aelterman et al., 2014, 2016; Reeve and Cheon, 2016). Since preservice teachers tend to exhibit CTB and believe that ASTB is difficult to implement (Barrett and Boggiano, 1988; Turner, 2010; Reeve and Cheon, 2016), a change in this belief is of particular importance. Reeve and Cheon (2016) argue that such changes can indicate the accommodation of new concepts. In their study as well as in the study by Aelterman et al. (2014), changes in physical education (PE) teachers’ beliefs about the ease of implementation and the effectiveness of ASTB after their intervention based on SDT could be found. When comparing the findings, it should be noted that the intervention in the studies by Aelterman et al. (2014, 2016) lasted 90 min longer and took place on one day, but contained the same elements as the intervention in the current study, including practical phases. Reeve and Cheon (2016) conducted an intervention that was three hours longer than the intervention in the current study and covered a larger period of time including the practice of the conveyed ASTBs in the PE teachers’ classes in the time between two sessions of the intervention. Such practical phases could not be implemented as part of the seminars we studied, as they do not include phases at schools, but might be included in future studies during the practical semester in the preservice teachers’ master’s degree.

The results regarding the preservice teachers’ belief about the effectiveness of ASTB depict a different picture than the results regarding the belief about the ease of implementation and the results of the studies by Aelterman et al. (2014, 2016) and Reeve and Cheon (2016). No significant differences were found in the comparison of the EG with both CGs (H3a, H3b). It might be that the preservice teachers in CG 2 also recognized the effectiveness of ASTB when they dealt with the concepts and assumptions behind SDT and the empirical findings concerning ASTB. However, this would not explain the non-significant difference between the EG and CG 1. In this case, it should be considered that in the pretest the preservice teachers in all groups already reported a high level of agreement to the items asking whether ASTB is an effective teaching practice. This strong agreement might have resulted in ceiling effects in the posttest.

A relatively strong agreement to the assessed beliefs about ASTB was also found in the study by Tan and Levesque-Bristol (2023), who examined the beliefs and intentions of novice and experienced preservice teachers. However, in comparison with the present study, it should be noted that no intervention was implemented to influence the investigated preservice teachers’ beliefs in their study (Tan and Levesque-Bristol, 2023). Aelterman et al. (2014, 2016) found a similar strong agreement to the belief about the effectiveness of ASTB and, as in our study, a comparatively lower expression of the belief about the ease of implementation of ASTB. However, changes in the belief about the effectiveness of ASTB after an intervention based on SDT could be found in their studies (Aelterman et al., 2014, 2016).

It is likely that the preservice and in-service teachers can easily imagine the effectiveness of ASTB based on their own experiences with autonomy-supportive and controlling settings and their own striving for autonomy. The preservice teachers’ strong agreement in our study might also reflect the fact that they were more advanced in their studies and may have already read or heard about the positive effects of autonomy support. Tan and Levesque-Bristol (2023) found no differences in the beliefs about ASTB in their comparison of novice and experienced preservice teachers, which contradicts this assumption. It should be noted, however, that the content of the teacher education program may differ among the countries in which the current study and Tan and Levesque-Bristol’s (2023) study were conducted.

### 6.3 Intention

Ceiling effects can also be assumed for the preservice teachers’ intention to apply ASTB. Here again, the preservice teachers’ agreement in all groups was already strongly pronounced in the pretest, as it is also reported in the study by Tan and Levesque-Bristol (2023). No significant differences were found between the EG and both CGs in our study (H4a, H4b). Although the investigated beliefs about ASTB were assumed to be predictors of the intention to apply this behavior (Reeve et al., 2014; Tan and Levesque-Bristol, 2023), a change in the preservice teachers’ belief about the ease of implementation did not seem to have led to a change in the preservice teachers’ intention to implement ASTB in their future teaching in the EG. In this context, it should be noted that there were ambiguous findings on the prioritization of individual beliefs regarding their importance in influencing one’s intention in previous studies. Whereas Reeve and Cheon (2016) claim that the belief about the ease of implementation is the strongest predictor for the intention to implement ASTB, findings by Großmann et al. (n.d.) suggest that the belief about the effectiveness thereof is the strongest one. In the study by Großmann et al. (n.d.), the belief about the ease of implementation of ASTB could not be confirmed as a predictor of the intention to apply it.

### 6.4 Beliefs about and intention to enact autonomy-supportive teaching behavior

Generally, it must be taken into account when comparing studies on interventions based on SDT that each intervention has unique characteristics, which means that comparisons across studies are usually difficult. In the comparison of previous studies, it can be stated that the effects of such interventions on student variables were more often investigated than on teacher variables (for an overview: Reeve and Cheon, 2021). The fact that teacher beliefs and intentions have received little attention in previous studies highlights the importance of their study in the context of teacher training. In addition, previous interventions often focus on in-service teachers and physical education (e.g., Aelterman et al., 2014; Reeve and Cheon, 2016). Positive beliefs about and intentions to enact ASTB should, however, be fostered as early as possible, preferably during teacher education at university. Moreover, science subjects should be increasingly focused on in future studies, where student motivation is declining and such interventions are urgently needed (Schiepe-Tiska et al., 2016; Scherrer and Preckel, 2019). Against the backdrop of these previous studies on beliefs about and intentions to enact ASTB, additional variables can be examined in future studies such as the autonomous orientation of participants (see Tan and Levesque-Bristol, 2023).

### 6.5 Limitations and implications

Despite our promising results, some limitations need to be addressed. First, we are not able to make statements about any possible behavioral changes of the preservice teachers in the EG with the data of the current study. Previous studies indicate that the use of new concepts is dependent on an individual’s beliefs about these concepts (e.g., Tillema and Knol, 1997; Reeve and Cheon, 2016). Thus, the positive impact of the intervention on the preservice teachers’ belief about the ease of implementation of ASTB can result in a change in their behavior. Moreover, the preservice teachers’ intention to apply ASTB, which was strongly pronounced in all investigated groups, can merely suggest what behavior they will exhibit in future classes. However, intention does not always predict behavior (Sheeran, 2002; Conner and Norman, 2022). For example, there is no prediction if the context does not offer the opportunity to implement the intended behavior (Sheeran, 2002). An investigation of possible changes in the preservice teachers’ behavior is therefore a significant desideratum for future studies (see, e.g., Aelterman et al., 2014; Reeve and Cheon, 2016; Cheon et al., 2020). Moreover, the effects of the intervention on the students taught by the preservice teachers who participated in the intervention need to be focused on in future studies (see, e.g., André et al., 2023).

It should also be taken into account that we cannot differentiate whether the additional time to practice the behaviors or the implementation of ASTB during the intervention or even both caused the differences between the EG and CG 2. Based on the variables in which the differences occurred (knowledge and belief about the ease of implementation), it can be assumed that the effects were mainly caused by the additional practice of the content. The additional effects of autonomy support during the intervention remain to be investigated in further studies, which could, for example, assess the preservice teachers’ quality of motivation during the intervention and the regular seminar. Regarding the practice of the content, it should be considered that an additional seminar session was needed to practice the measures. Providing this additional time might not be possible in all teacher-training seminars at university. To address this problem, interventions could implement both face-to-face elements in the seminar and web-based elements for independent study (see Tilga et al., 2021). In a study by Tilga et al. (2021), this type of combined intervention was found to have the greatest effects on PE teachers’ ASTB and CTB.

In this regard, it has to be mentioned that CTB was not investigated in our study. Future studies could include beliefs about CTB and intentions to implement it. It might be that despite the changes regarding ASTB, positive beliefs about the ease of implementation and effectiveness of CTB as well as intentions to enact this behavior are strongly pronounced before and after the intervention. Regarding intentions, however, it should be noted that for some of the autonomy-supportive measures, such as providing choice, the intention can only be either autonomy-supportive (providing choices) or controlling (not providing choices).

With regard to the two other basic psychological needs, future interventions could incorporate relatedness and competence support to encourage these behaviors in the classroom as well. To design such interventions, the classification system by Ahmadi et al. (2023) could be used. In this system, motivational behaviors were rated and categorized in terms of their impact on the basic needs for autonomy, competence, and relatedness (Ahmadi et al., 2023). Regarding ASTBs, it should be noted that these behaviors might be considered as multidimensional in future interventions, for example, based on Stefanou et al.’s (2004) categorization, which includes organizational, procedural, and cognitive autonomy support (see also Tilga et al., 2021). In addition to expanding and differentiating the behaviors conveyed in the intervention of the current study, interventions to teach other theories of motivation could be designed (e.g., expectancy-value theory: Wigfield and Eccles, 2000). Thus, it could be investigated whether the interventions based on other theories of motivation show similar effects as the current intervention based on SDT. Moreover, the intervention could be applied as further training for in-service teachers (see also Mittag et al., 2009) and university lecturers, which is currently being implemented.

Additionally, it should be noted that despite the implementation of CG 1, we have no way of knowing if the preservice teachers in EG and CG 2 did not learn any content on SDT and ASTB in other seminars in the interim. During the time of the intervention, all students took part in the seminars dealing with the practical semester in educational sciences and their second subjects, which were very diverse (see Section 4.1). However, given that the intervention only covers a short period of time, learning and practicing the contents of the intervention in other seminars during the time of the intervention is not likely. In future studies, the posttest could additionally ask what content is covered in the seminars in educational sciences and the second subject during the time of the intervention to test these assumptions.

Lastly, we would like to point out that the strong agreement that we found for the intention to apply ASTB, and for the belief about the effectiveness of ASTB as well, in the pretest has to be viewed positively. The belief that could still hinder the actual application of ASTB is the one about the ease of implementing it (Reeve et al., 2014; Reeve and Cheon, 2016), which was only slightly pronounced among the preservice teachers. Particularly during stressful times such as in-service teacher training or exercising a full-time teaching position with a high teaching load, teachers will probably choose those measures that they believe to be easy to implement in their class. Our findings indicate that interventions should focus on this belief, which seems to be positively affected by practical elements. To give more space to the practical elements in seminars, the acquisition of the content could already take place before the seminar with web-based content in independent study (see Tilga et al., 2021). Besides the practice and discussion of the content, students could be given space to identify their own beliefs, recognize the origin of their beliefs, and reflect on them.

## 7 Conclusion

The integration of practical phases at school into university teacher training is demanded to support preservice teachers’ development of teaching skills (see Grossman et al., 2009; Philip et al., 2019). The current quasi-experimental study shows that university seminars can also offer opportunities to practice such skills. In our study, such phases for practicing the content seem to be the missing element in the regular teacher training seminars when it comes to equipping preservice teachers with effective and easily implementable options to counteract students’ declining motivation (Gillet et al., 2012; Scherrer and Preckel, 2019). Specifically, the results of our study show that the developed intervention based on SDT had a positive impact on preservice teachers’ knowledge and beliefs about ASTB as well as their intention to apply it in the future. Whether the preservice teachers would actually use ASTB in their classes must be the subject of further investigation.

## Data availability statement

The raw data supporting the conclusions of this article will be made available by the authors, without undue reservation.

## Ethics statement

The studies involving humans were approved by Ethics Committee of Bielefeld University. The studies were conducted in accordance with the local legislation and institutional requirements. Written informed consent for participation was not required from the participants or the participants’ legal guardians/next of kin because the research involves no more than minimal risk to the subjects and involves no procedures for which written consent is normally required outside of the research context. Moreover, no personal data were collected that would have required written consent according to the guidelines in the country of the study.

## Author contributions

NG: Conceptualization, Formal analysis, Investigation, Methodology, Writing – original draft. SF: Conceptualization, Writing – review & editing. MW: Conceptualization, Writing – review & editing.
